# New potential beneficial effects of actein, a triterpene glycoside isolated from *Cimicifuga* species, in breast cancer treatment

**DOI:** 10.1038/srep35263

**Published:** 2016-10-12

**Authors:** Grace Gar-Lee Yue, Sida Xie, Julia Kin-Ming Lee, Hin-Fai Kwok, Si Gao, Yin Nian, Xiao-Xiao Wu, Chun-Kwok Wong, Ming-Hua Qiu, Clara Bik-San Lau

**Affiliations:** 1 Institute of Chinese Medicine, The Chinese University of Hong Kong, Shatin, New Territories, Hong Kong; 2 State Key Laboratory of Phytochemistry and Plant Resources in West China, The Chinese University of Hong Kong, Shatin, New Territories, Hong Kong; 3 State Key Laboratory of Phytochemistry and Plant Resources in West China, Kunming Institute of Botany, Chinese Academy of Sciences, Kunming 650201, China; 4 Department of Chemical Pathology, The Chinese University of Hong Kong, Shatin, New Territories, Hong Kong

## Abstract

Actein is a triterpene glycoside isolated from the rhizomes of *Cimicifuga foetida* (Chinese herb “shengma”) which could inhibit the growth of breast cancer cells. Nevertheless, the effect of actein on angiogenesis, which is an essential step for tumor growth and metastasis, has never been reported. Hence, this study aimed to investigate the *in vitro* and *in vivo* effects of actein on angiogenesis using human microvascular endothelial cells (HMEC-1), matrigel plug and tumor-bearing mouse models. Our results showed that actein significantly inhibited the proliferation, reduced the migration and motility of endothelial cells, and it could suppress the protein expressions of VEGFR1, pJNK and pERK, suggesting that JNK/ERK pathways were involved. *In vivo* results showed that oral administration of actein at 10 mg/kg for 7 days inhibited blood vessel formation in the growth factor-containing matrigel plugs. Oral actein treatments (10–15 mg/kg) for 28 days resulted in decreasing mouse 4T1 breast tumor sizes and metastasis to lungs and livers. The apparent reduced angiogenic proteins (CD34 and Factor VIII) expressions and down-regulated metastasis-related *VEGFR1* and *CXCR4* gene expressions were observed in breast tumors. Our novel findings provide insights into the use of actein for development of anti-angiogenic agents for breast cancer.

*Cimicifuga* species have been used for centuries as traditional medicinal herbs in North American, Asia and Europe. *C. racemosa* (perennial black cohosh) was used by Native Americans for anti-inflammation and alleviating menopausal symptoms^1^. In Asia, other *Cimicifuga* species are reported to possess anti-osteoporosis, anti-viral, anti-diabetic, anti-malarial and vasoactive properties^2^, and could be used as antipyretic and analgesic agents^3^. According to Chinese Pharmacopoeia, the dried rhizome of *C. dahurica* (Turcz.) Maxim., *C. foetida* L. and *C. heracleifolia* Kom. are defined as herb Cimicifugae Rhizoma or “Shengma”, with heat-clearing and detoxifying effects^4^. In the past decades, over hundreds of triterpene glycosides/cycloartane triterpenoids have been isolated from the roots and rhizomes of *Cimicifuga* species by different research groups^5^,^6^,^7^. Previous *in vitro* studies demonstrated that the growth inhibitory activity on breast cancer cells of *C. racemosa* (black cohosh) extracts was related to the triterpene glycoside composition^8^,^9^. Several cycloartane triterpenoids isolated from *C. yunnanensis* were also shown to induce apoptosis of breast cancer cells via p53-dependent mitochondrial pathway^10^. Most recent findings demonstrated that cycloartane triterpenoids isolated from *C. foetida* could inhibit Raf/MEK/ERK signaling pathway and Akt phosphorylation in breast cancer MCF-7 cells^11^, as well as suppress TNFα-induced IKKα/β and IKBα phosphorylation, and nuclear factor (NF)-κB downstream target gene expression in triple-negative breast cancer cells^12^.

The purified triterpene glycoside actein (*β*-D-xylopyranoside, Fig. 1A) from black cohosh was shown to be selective for human breast cancer cells^8^,^13^ and able to synergize at low concentrations with different classes of chemotherapy agents to inhibit cancer cell growth^14^,^15^, as well as induced calcium release and modulated the NF-κB and Ras/Raf/mitogen-activated protein kinase/ERK kinase (MEK) pathways^16^. On the other hand, actein was demonstrated recently to promote the function of osteoblastic MC3T3-E1 cells and protect against methylglyoxal-induced oxidative damage in these cells^17^,^18^. Animal studies also showed that actein could alter the expression of cholesterol and fatty acid biosynthetic genes, p53 pathway genes in rats^19^. Nevertheless, the activities of actein on angiogenesis and tumor growth in tumor-bearing animal models have never been reported.

In fact, the *in vivo* anti-tumor activities of extracts or active components from *Cimicifuga* species have been reported, such as extracts of *C. racemosa* in breast cancer rat model^20^, prostate cancer mouse model^21^ and total glycosides from *C. dahurica* in hepatoma-bearing mice^22^,^23^. However, controversial findings were also observed in transgenic mice expressing c-erbB2, in which extract of *C. racemosa* increased metastatic mammary cancer^24^. Similarly, there was controversy on the clinical use of *C. racemosa* and its impact on breast cancer risk as well as the chemopreventive and anticancer potential of this herb^25^,^26^,^27^. In the present study, the anti-tumor activities of actein, which could be isolated from both *C. racemosa*^13^ and *C. foetida*^28^,^29^, would be investigated in details.

Breast cancer has heterogeneous nature and can be categorized to different molecular subtypes based on gene expression profiling^30^. It is also known to be metastatic at its latter stages, during which angiogenesis plays an important role. Angiogenesis is essential for tumor growth and it is believed that blocking the expanding network of newly formed capillaries could be a strategy to arrest tumor growth and metastasis^31^. Different responsible angiogenic pathways among different molecular subtypes of breast cancer were investigated in preclinical and clinical studies over the last decade^32^. Several humanized antibodies targeting these pathways have been testified for their efficacies^33^, such as the anti-vascular endothelial growth factor (VEGF)-A antibody bevacizumab (Avastin^®^)^34^, anti-VEGFR2 antibody ramucirumab (IMC-1121B)^35^, and aflibercept (VEGF-Trap)^36^. Many clinical trials on the tyrosine kinases inhibitors (TKIs), targeting the intracellular domain of the VEGFRs and blocking their catalytic function, are currently in progress. TKIs such as sunitinib (Sutentw^®^), sorafenib (Nexavarw^®^), pazopanib (Votrientw^®^) and axitinib (Inlytaw^®^) have been shown to have antiangiogenic effects in breast cancer patients^32^,^33^. Nevertheless, searching for new antiangiogenic agents, especially from natural source including herbal medicines, draws preclinical researchers’ interest. Some chemopreventive molecules isolated from natural products, including taxol^37^, curcumin^38^, deoxybouvardin^39^, and aromatic-turmerone^40^ are also demonstrated to inhibit angiogenesis in preclinical models. Being reported as an anti-tumor natural compound, actein has not been studied for its activity on angiogenesis and in breast tumor-bearing animal models.

We herein reported for the first time that actein, the active compound found in *Cimicifuga* species, possessed anti-angiogenic and immunomodulatory effects in a murine breast tumor-bearing model. The VEGFR1, c-Jun N-terminal kinase (JNK) and extracellular signal-regulated kinase (ERK) signaling pathways were involved in actein’s anti-angiogenic activities, which might subsequently inhibit the orthotopic tumor growth and metastasis of tumor cells in mice. Besides, the beneficial role of oral administered actein in immune responses of breast tumor-bearing mice was also firstly revealed in the present study.

## Results

### Actein inhibited cell proliferation and cell migration in human endothelial cells

**Figure 1:**
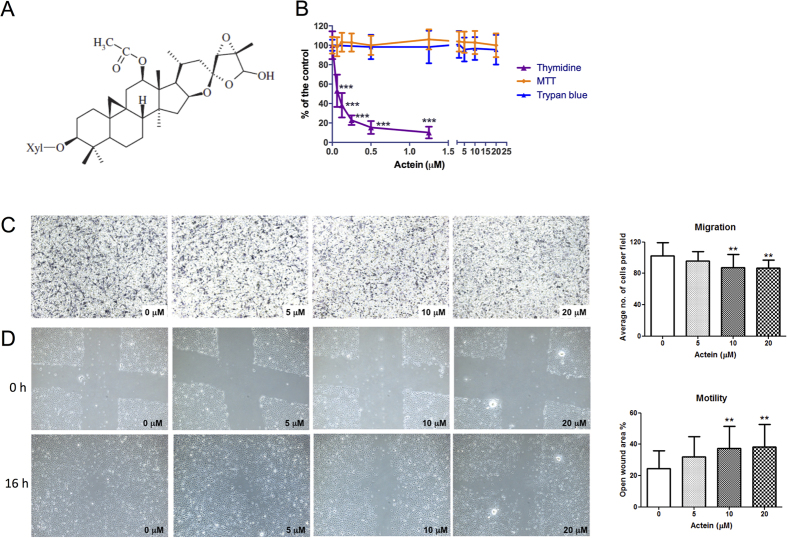
Effects of actein on cell proliferation, migration and motility of HMEC-1 cells. (**A**) Chemical structure of actein. (**B**) Cells were treated with different concentrations of actein for 48 hours, then cytotoxicity and cell proliferation was determined by MTT, trypan blue and [methyl-^3^H] thymidine incorporation assays, respectively. Results are expressed as the mean % ratio of optical density or count per minute in treated and vehicle-treated control wells (mean ± SD of 3 independent experiments with 5 wells each). (**C**) Representative photomicrographs showing the migrated and stained HMEC-1 cells on the lower side of membranes in the presence or absence of actein. Quantification of cell migration in modified Boyden chambers was shown. Results are expressed in number of cells per field (mean + SD of 4 independent experiments). (**D**) Representative photomicrographs showing the cells migrated across the scratch wound in the presence or absence of actein after 16 hours of incubation. Quantification of wound-induced cell motility in HMEC-1 was shown. Results are expressed as the mean open wound area (mean + SD of 3 independent experiments). Differences among the treated and vehicle-treated control groups were determined by one-way ANOVA with Tukey’s *post-hoc* test. **p < 0.01, ***p < 0.001 as compared to control group.

The cytotoxicity of actein on human endothelial cells HMEC-1 was determined using MTT assay after 48 hours of incubation. As shown in Fig. 1B, actein (0.625–20 μM) did not cause significant cytotoxicity in HMEC-1 cells. Results from trypan blue assay also showed that actein at tested concentration did not affect the viability (viable cell numbers) of HMEC-1 cells. While the cells treated with actein assessed by ^3^H-thymidine incorporation assay, the cell proliferation was significantly inhibited in a concentration-dependent manner. The concentration producing 50% growth inhibition (IC_50_) of actein was 0.065 μM. The presence of vehicle, 0.5% DMSO, did not affect the cell proliferation of endothelial cells (data not shown). The subsequent cell assays were performed using actein (<20 μM) so that the inhibitory activities observed were not due to cytotoxicity.

The effects of actein treatment on endothelial cell migration were evaluated in modified Boyden chambers. After 6 hours of incubation, cells migrated from the upper chamber to the lower chamber through the membrane as the chemoattractant gradient formed by 10% (v/v) FBS-containing culture medium in lower chamber. In the presence of actein (10 and 20 μM), the migrated cell numbers decreased in a concentration-dependent manner, thereby suggesting the inhibitory effect of actein on the migration of HMEC-1 cells (Fig. 1C). Furthermore, actein was shown to reduce the motility of endothelial cells in scratch wound assay as the open wound area was increased in actein-treated wells (Fig. 1D). The inhibitory effect of actein on cell motility was in a concentration dependent manner. The open wound areas in 10–20 μM actein-treated wells were significantly greater than those of vehicle-treated wells (*p* < 0.01).

### Actein suppressed the protein expressions of VEGFR1 and downregulated JNK, ERK signaling pathways in endothelial cells

**Figure 2:**
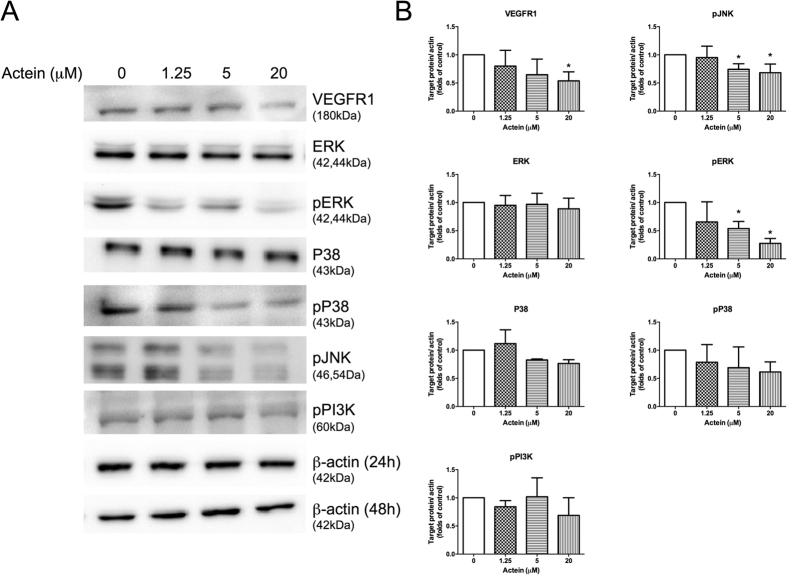
Effects of actein on expressions of VEGFR1 and signaling kinases in HMEC-1 cells. Western blot analyses on P38, pP38, ERK, pERK, pJNK, pPI3K expressions in actein-treated HMEC-1 cells for 24 hours (or 48 hours for VEGFR1) were performed. (**A**) Immunoblotting was performed 3 times using independently prepared cell lysates and the representative blots were shown. (**B**) The histograms showed the quantified results of protein levels, which were adjusted with corresponding β-actin protein levels and expressed as fold of control (mean fold of control + SD from 3 independent experiments). Differences among the treated and vehicle-treated control groups were determined by one-way ANOVA with Tukey’s *post-hoc* test. *p < 0.05 as compared to control group.

To examine the effects of actein on VEGFR1 expression and to explore the underlying mechanism of action, protein of actein-treated HMEC-1 cells were collected for Western blot analysis. As shown in Fig. 2A, actein decreased the expressions of VEGFR1 in a concentration-dependent manner in HMEC-1 cells, with 20 μM the most potent. The phosphorylation of JNK and ERK were significantly reduced by actein at 5 and 20 μM (*p* < 0.05). The levels of pP38 MAPK and P38 were slightly reduced in actein-treated HMEC-1, whereas the changes of pPI3K and ERK were not apparent after actein treatment (Fig. 2B). The significant reduction of actein on the phosphorylation of JNK and ERK may subsequently inhibit cell migration and motility of the endothelial cells (Fig. 2B).

### Orally administered actein inhibited blood vessel formation in mouse matrigel plug model

**Figure 3:**
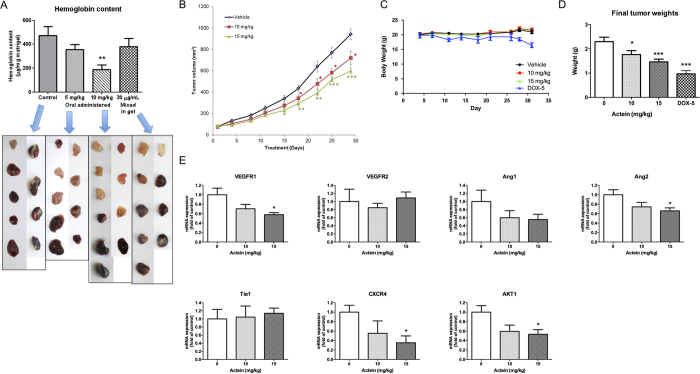
Effects of actein on angiogenesis and tumor growth in mouse models. (**A**) Upper: Hemoglobin content of matrigel plugs from different treatment groups (n = 9-10). Lower: The photos of matrigel plugs excised from mice of different treatment groups 7 days after inoculation. (**B–E**) 4T1 tumor-bearing mice were treated with actein (10 or 15 mg/kg) or vehicle for 4 weeks. The volumes of tumors (**B**) and body weights (**C**) were recorded and expressed as mean ± SEM (n = 12). (**D**) The final tumor weights after actein or vehicle treatments. (**E**) Quantitative RT-PCR analyses of *VEGFRs, Ang1, Ang2, Tie1, CXCR4, AKT* mRNA expressions in tumors excised from different treatment groups. Tumor tissues were collected for RNA extraction. Data were normalized to corresponding human *beta-2 microglobulin (B2M*) expressions in tumors of vehicle-treated group. mRNA expressions results are expressed as fold of control (mean fold of control + SEM from 8 mice each group). Differences among the treated and vehicle-treated control groups were determined by one-way ANOVA with Tukey’s *post-hoc* test. *p < 0.05, **p < 0.01, ***p < 0.001 as compared to control group.

To further verify the *in vivo* inhibitory effect of actein on new blood vessel formation (i.e. angiogenesis), the mouse matrigel plug assay was performed. Seven days after the plugs were inoculated, plugs in untreated control group exhibited red color indicating that new blood vessels formation occurred (Fig. 3A, lower panel), whereas some of the plugs mixed with actein (30 μg/mL gel or equivalent to 44.3 μM) did not show red color (e.g. the first one in the photo). The blood vessel formation might be reduced by actein *in situ*. In contrast, the plugs from mice orally administered with actein (5 or 10 mg/kg) for 7 days were of light red or pale pink color indicating that fewer blood vessels were formed. Furthermore, the hemoglobin content in the plugs was measured to quantify the extent of angiogenesis. As shown in histogram of Fig. 3A, the hemoglobin contents in the plugs of mice treated with 10 mg/kg actein (187.1 μg hemoglobin/mg matrigel) were significantly lower than those of untreated control mice (473.6 μg hemoglobin/mg matrigel) (*p* < 0.01). Results suggested that the inhibitory effects on blood vessel formation could be achieved by oral administration of actein to mice.

### *In vivo* anti-angiogenic and anti-tumor activities of actein in breast tumor-bearing mice

Mouse syngeneic 4T1 mammary tumors were formed at the mammary fat pad of immunocompetent female BALB/c mice in order to investigate the *in vivo* activities of actein on angiogenesis and tumor growth. Tumor-bearing mice were orally administered with actein 10 or 15 mg/kg for 4 weeks. The tumor volumes of actein-treated mice were significantly lower than those of untreated control mice since the 18^th^ day of treatment (*p* < 0.05, Fig. 3B). During the treatment, no body weight loss was observed in all groups and there was no difference among them (Fig. 3C). In doxorubicin treated group (positive control group), the tumor weights were significantly decreased when compared to untreated vehicle group. However, the body weights of tumor-bearing mice were significantly decreased after doxorubicin treatment but not after actein treatment. At the end of experiment, excised tumors were weighed as shown in Fig. 3D. The tumor weights of actein treatment (10 and 15 mg/kg) groups were significantly decreased by 21.2% and 36.4% when compared with control group, respectively.

The mRNA expressions of several angiogenic genes (*VEGFR1, VEGFR2, Ang1, Ang2* and *Tie1*) in the tumors were determined by real-time PCR analysis. Results showed that the expressions of *VEGFR1* and *Ang2* were significantly downregulated by 15 mg/kg actein treatment (Fig. 3E). As angiogenesis is also crucial for tumor metastasis, the mRNA expressions of metastasis-related molecules CXCR4 and AKT were also examined in tumors. Tumors from actein-treated mice were found to have lower expression levels than those from control mice (Fig. 3E).

**Figure 4:**
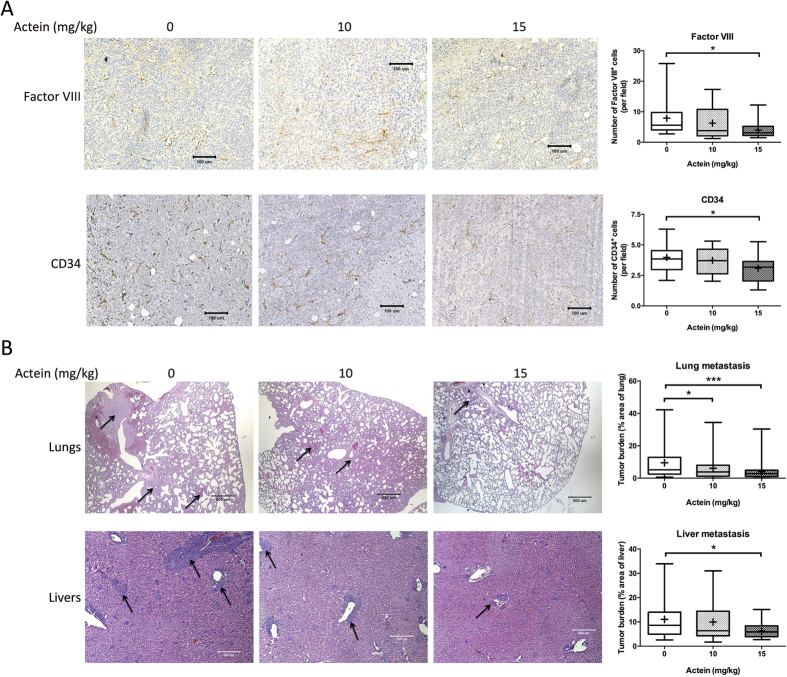
Histopathology of tumors, lungs and livers of tumor-bearing mice after actein treatments. (**A**) Endothelial cells in the tumor sections were assessed using Factor VIII and CD34 immunohistochemical analysis. Representative photomicrographs of magnification 40X showing the endothelial cells stained with anti-Factor VIII or anti-CD34 antibodies in brown. Quantification of Factor-VIII or CD34 positive-stained cells in tumor sections was conducted in a blinded manner. Results were expressed in box and whisker plots of 12 mice each group. (**B**) The paraffin-embedded sections of the lungs and livers were photographed and used to measure metastatic loci area and total lung or liver area. The histograms showed the tumor burden in lungs and livers according to the tumor area as a percentage of whole lung or liver area per group. Representative H&E-stained sections of lungs and livers from different groups with arrows showing the metastatic loci. Results were expressed in box and whisker plots of 12 mice each group. Differences among the treated and vehicle-treated control groups were determined by one-way ANOVA with Tukey’s *post-hoc* test. *p < 0.05, **p < 0.01, ***p < 0.001 as compared to control group.

In order to further confirm the anti-angiogenic activities of actein in breast tumor-bearing mice, immunohistochemical analysis in tumor sections have been performed. Figure 4A showed the formed neovasculatures in tumor sections stained by Factor VIII- or CD34-specific antibodies. The numbers of positive-stained cells (brown spots in photos) were found to be decreased in actein-treated mice tumors. Results suggested that oral administration of actein in tumor-bearing mice may inhibit the angiogenesis in the tumors, which then reduce the blood supply for the tumors and suppress the tumor growth.

In addition, the anti-metastatic activities of actein were observed in lungs and livers of tumor-bearing mice treated with actein. Parafilm-embedded sections of lungs and livers were assessed for the tumor burden in a blinded manner. As shown in the photos of Fig. 4B, large metastatic loci were found in vehicle-treated control group, while the area of metastatic loci was decreased in actein-treated group in a dose-dependent manner (Fig. 4B). Tumor burden in lungs and livers from 15 mg/kg actein-treated group was found to decrease by 57.5% and 43.4%, respectively, when compared with vehicle-treated group (Fig. 4B).

### Actein regulated immune responses in breast tumor-bearing mice

**Figure 5:**
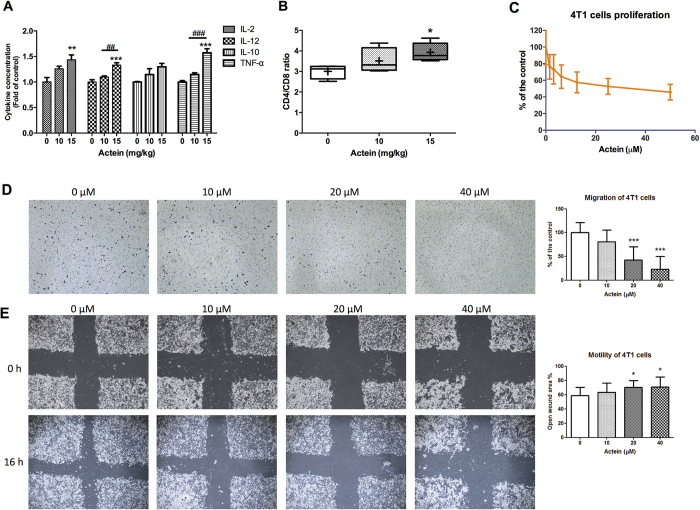
Effects of actein on cytokine profiles and T-lymphocyte subset distribution of tumor-bearing mice and on 4T1 cell proliferation and migration. Spleens of tumor-bearing mice were excised for lymphocyte isolation as described in methodology section. Lymphocytes were incubated for 24 hours and the culture supernatants were collected. Cytokines concentrations of (**A**) IL-2, IL-10, IL-12, TNF-α were specifically determined by ELISA. Results were expressed as fold of control (mean fold of control + SEM from 6 mice each group). Another portion of spleen lymphocytes were with T-lymphocyte subset antibody cocktail and the number of stained cells for CD3, CD4 and CD8 were quantified by fluorescence activated cell sorting and were depicted here as the ratio of CD4^+^ and CD8^+^ cells per 20,000 viable cells. (**B**) Results were expressed in box and whisker plots of 6 mice each group. (**C**) 4T1 cells were treated with different concentrations of actein for 48 hours, then cell proliferation was determined by [methyl-^3^H] thymidine incorporation assays. Results are expressed as the mean % count per minute in treated and vehicle-treated control wells (mean ± SD of 3 independent experiments with 5 wells each). (**D**) Representative photomicrographs showing the migrated and stained 4T1 cells on the lower side of membranes in the presence or absence of actein. Quantification of cell migration in modified Boyden chambers was shown. Results are expressed in number of cells per field (mean + SD of 4 independent experiments). (**E**) Representative photomicrographs showing the cells migrated across the scratch wound in the presence or absence of actein after 16 hours of incubation. Quantification of wound-induced cell motility in 4T1 was shown. Results are expressed as the mean open wound area (mean + SD of 3 independent experiments). Differences among the treated and vehicle-treated control groups were determined by one-way ANOVA with Tukey’s *post-hoc* test. **p < 0.01, ***p < 0.001 as compared to control group. *p < 0.05, **p < 0.01, ***p < 0.001 as compared to control group. ^##^*p* < 0.01, ^###^*p* < 0.001 as compared among groups.

The immunomodulatory activities of actein in tumor-bearing immunocompetent mice were demonstrated here. The cytokines levels in culture supernatant of spleen lymphocytes isolated from mice treated with vehicle or actein were determined by ELISA (BD Biosciences, NJ, USA). The productions of Th1-cytokines (IL-2, IL-12 and TNF-α) were increased in actein-treated group in a dose-dependent manner (Fig. 5A). Furthermore, the CD4/CD8 ratio in the peripheral blood lymphocytes was significantly increased in 15 mg/kg actein treatment group when compared with vehicle-treated group (Fig. 5B). These findings suggested that the immunomodulatory activities of actein in tumor-bearing mice may also contribute to its anti-tumor and anti-metastasis effects.

On the other hand, the *in vitro* effects of actein on the proliferation, migration and motility of 4T1 cells were further evaluated. As shown in Fig. 5C–E, actein exerted inhibitory effect in these cellular activities, suggesting that actein would also play role in the anti-metastatic effect in tumor-bearing mice.

## Discussion

Actein is a bioactive triterpene glycoside isolated from *Cimicifuga* species (such as *C. racemosa* and *C. foetida*) and has been demonstrated for its inhibitory activities on the growth of breast cancer cells^8^,^13^ as well as of osteoblastic cells^17^,^18^. Limited information regarding the *in vivo* effects of actein on tumor growth, especially breast cancer, has ever been shown. Though the efficacies of extracts of other *Cimicifuga* species in tumor-bearing models were reported, the findings were controversy^20^,^21^,^22^,^23^,^24^. In the present study, the efficacies of actein on angiogenesis, breast tumor growth and metastasis in an immunocompetent mouse model were revealed for the first time. The mechanistic studies suggested the involvement of VEGFR1 as well as the activation of JNK and ERK signaling pathways for the action. These findings provide insights into the use of actein or herbs containing actein for cancer adjuvant therapy or development of anti-angiogenic agents.

In our endothelial cell model, actein was found to inhibit HMEC-1 cell proliferation without cytotoxicity, with IC_50_ value at 65 nM, which was the lowest among other derivatives tested in endothelial cells (data not shown). Hence, the activities of actein on endothelial cells migration and motility were examined and were shown to be inhibitory. The derivative of actein, deoxyactein, also possessed similar suppressive activities in endothelial cells with higher effective doses when compared with actein^41^. Meanwhile, actein was recently shown to inhibit proliferation and migration of osteosarcoma cells^42^. From our Western blot results, actein could apparently suppress the expressions of VEGFR1, pJNK and pERK in HMEC-1 cells. The decreases in pPI3K and pP38 MAPK expressions were also observed in actein-treated HMEC-1 cells. Such changes of signaling molecules may be responsible for the inhibition of proliferation and migration of endothelial cells^43^,^44^. Previous studies showed that other cycloartane triterpenoids isolated from *C. yunnanensis* and *Homonoia riparia* exerted anti-angiogenic activities in HUVEC^6^,^45^.

The anti-angiogenic effects of actein were further confirmed using *in vivo* mouse models. In the matrigel plug model, the blood vessel formation in term of hemoglobin content in the plugs was significantly reduced by oral administered actein to mice. Whereas the matrigel mixed with actein exhibited mild suppressive effect on blood vessel formation in the plugs. It was possible that the metabolites of actein after ingestion would contribute to the inhibitory effect on angiogenesis. The pharmacokinetics of actein in Sprague-Dawley rats have been reported that after gastric intubation of 35.7 mg/kg actein, the serum level of actein peaked at 2.4 μg/mL at 6 hours^19^. Nonetheless, details of metabolites of actein, or even for cycloartane triterpenoids, have seldom been reported. In our *in vivo* models, mice were orally administered with actein which was expected to be absorbed and/or metabolized and then distributed in the whole body, including tumors. The promising *in vivo* activities showed here warrant further pharmacokinetics studies for these bioactive compounds.

Furthermore, the *in vivo* anti-angiogenic activities of actein were verified in immunocompetent breast tumor-bearing mice, in which tumor growth, new blood vessels formation in tumors as well as lung and liver metastasis were reduced after actein treatments. Angiogenesis plays an important role in cancer growth, infiltration and metastasis^31^. Antiangiogenic drugs interfere with this process and could be responsible for inducing tumor dormancy by blocking the expanding network of newly formed capillaries and revealed a potential efficacy in breast cancer^32^. Here, we showed that actein treatment suppressed the mRNA expressions of *VEGFR1, CXCR4* and *AKT1* in tumor tissues. VEGFR-1 and VEGF expressions were higher in breast cancer tumor when compared to surrounding tissues^46^. VEGF receptors may not only modulate angiogenesis through the ERK or AKT pathways, but also directly influence the growth of VEGF receptor expressing tumors^47^. Besides, invasive and *in situ* breast cancers express many angiogenic factors (e.g. *Ang1* and *Ang2*) and other metastasis-related molecules (e.g. *CXCR4*) throughout all tumor stages^48^. The downregulation of these genes in breast tumors (which consisted of tumor cells, endothelial cells and other cell types) by actein might account for its anti-angiogenic and anti-metastatic effects. Moreover, the protein and mRNA expressions of VEGFR1 were suppressed by actein. The binding ability/interaction between actein and VEGFR1 should be further studied by molecular docking experiments.

Besides, a novel finding of the present study is that the immune responses of breast tumor-bearing mice were interfered by actein treatments. The cytokine profiles and T lymphocyte subset distribution of spleen lymphocytes were altered in actein-treated mice. Previous *in vivo* studies on *Cimicifuga* species used athymic (nude) mice^21^,^22^,^24^, which lack of B- and T-cell function. Numerous studies advocated that the immune system plays an integrative role in cancer biology, both controlling tumor growth and mediating the eradication of disease^49^. The upregulation of inhibitory immune checkpoint pathways, e.g. PD-1 pathway, tumor cells and immune cells within the tumor microenvironment further inhibit the activation of tumor antigen–specific T cells^50^. The shifted cytokine profiles of T cells and CD4/CD8 ratio after actein treatments might also play role in regulating the tumor progression. Lee and Choi demonstrated that actein suppressed the production of TNF-α and oxidative stress in the presence of a mitochondrial inhibitor^17^. The anti-inflammatory activity of cimiracemate A isolated from *C. racemosa* via NFκB and MAPK pathways in human macrophages were also reported^51^. Recent study reported that cycloartane glycosides from *C. foetida* showed significant Wnt signaling pathway inhibitory activity in luciferase reporter gene assay^52^. Nonetheless, the immune responses of tumor-bearing mice after actein treatments were firstly demonstrated in the present study.

Last but not least, being the allied species of *C. racemosa* (black cohosh), *C. foetida* (shengma) contains actein and many other bioactive cycloartane triterpenoids^6^,^28^. The new potential application of this herb for angiogenesis-dependent diseases warrants further investigation. On the other hand, controversy is surrounding the use of hormone replacement therapy and black cohosh for breast cancer patients^27^. Scientific research on the combined use of herbs with conventional medicines in various pathological conditions would be essential for future development of complementary and alternative medicines, such as Chinese medicines.

In conclusion, actein was shown to possess anti-angiogenic effects on human endothelial cells, which may act through inhibition of the JNK/ERK signaling and VEGFR1 activation. Besides, the results of *in vivo* studies using mouse models suggested that actein treatment significantly inhibited the blood vessel formation and in turn exerted anti-tumor and anti-metastatic effects. The findings from this study revealed the potential of actein for future breast cancer adjuvant therapy.

## Materials and Methods

### Chemicals and reagents

The human microvascular endothelial cells (HMEC-1) and mouse breast tumor cells (4T1) were purchased from American Type Culture Collection (MD, USA). RPMI 1640 medium, fetal bovine serum (FBS), penicillin-streptomycin, trypsin-EDTA, Trizol, recombinant human VEGF were obtained from Life Technologies (NY, USA). MCDB 131 medium, epidermal growth factor, basic human fibroblast growth factor (bFGF), gelatin, glutamine, hematoxylin & eosin, hydrocortisone, 3-(4,5-dimethylthiazol-2-yl)-2,5-diphenyl-tetrazolium bromide (MTT) and Drabkin’s reagent were obtained from Sigma-Aldrich (MO, USA). Basement membrane matrix Matrigel (growth factor reduced) was obtained from BD Biosciences (NJ, USA). The antibodies for Western blot, P38 MAPK (Thr180/Tyr182) (#9212), Phospho-P38 MAPK (Thr180/Tyr182) (#9215), p44/42 MAPK (Erk1/2) (137F5) (#4695), Phospho-p44/42 MAPK (Erk1/2) (Thr202/Tyr204) (#4377), Phospho-PI3 Kinase p85 (Tyr458)/p55 (Tyr199) (#4228), Phospho-SAPK/JNK (Thr183/Tyr185) (#4668), VEGFR1 (#2893) were obtained from Cell Signaling (MA, USA), while β-actin (#A5316) was purchased from Sigma-Aldrich (MO, USA) and the secondary antibodies HRP-Goat Anti-Rabbit IgG (#65-6120) and HRP-Goat Anti-Mouse IgG (#62-6520) were obtained from Life Technologies (NY, USA). The antibodies for immunohistochemistry, CD34 (MEC14.7) was from abcam (UK), von Willebrand factor (Factor VIII) and rat-on-mouse HRP-polymer kit were from Biocare (CA, USA). Transwell polycarbonate cell culture inserts (6.5 mm diameter, 8 μm pore size) were from Costar (MA, USA). The *in situ* cell death POD kit was from Roche (Germany). [Methyl-^3^H]-thymidine and unifilters were obtained from PerkinElmer (MA, USA). Real-time PCR reagent iTaq Fast SYBR Green Supermix was obtained from Bio-Rad (Hong Kong).

### Plant materials

The rhizomes of *C. foetida* were collected in 2014 from Daju County, Lijiang Prefecture, Yunnan Province and identified by Prof. Pei Sheng-Ji, Kunming Institute of Botany, Chinese Academy of Sciences. A voucher specimen (KUN No. 20100906) has been deposited at the State Key Laboratory of Phytochemistry and Plant Resources in West China, Kunming Institute of Botany, Chinese Academy of Sciences, China.

### Extraction and isolation of actein

The air-dried and powdered rhizomes of *C. foetida* (12 kg) were refluxed with MeOH (3 × 20 L) for 5 hours. The residue was extracted successively with petroleum ether, EtOAc and *n*-BuOH. The EtOAc (900 g) extract was subjected to silica gel column chromatography, eluted with CHCl_3_−MeOH (CHCl_3_, 100 : 1, 50 : 1, 20 : 1, 5 : 1) to give five fractions (Fr. I−Fr. V). Fr. IV (350 g) was further divided into five sub-fractions (Fr. IV.1–5) after performing silica gel column chromatography, eluted with CHCl_3_−acetone (10 : 1). After recrystallization from Fr. IV.5, further purification was conducted on semi-preparative HPLC (Agilent, Zorbax, SB-C18, 5 μM, 9.4 mm × 250 mm; eluted with CH_3_CN-H_2_O, 42:58; retention time: about 12 min) to obtain pure actein (3 g).

Actein was dissolved in dimethylsulfoxide (DMSO) and stored at −20 °C and reconstituted in appropriate media prior to the experiments. The vehicle control cultures received the vehicle solvent (0.5% v/v DMSO).

### Cell culture and proliferation assays

HMEC-1 cells were maintained in MCDB 131 medium containing 2 mM glutamine, 1 μg/mL hydrocortisone and 10 ng/mL epidermal growth factor. Mouse breast tumor 4T1 cells were maintained in RPMI 1640. All media were supplemented with 10% v/v heat-inactivated FBS, 100 units/mL penicillin-streptomycin. The cells were incubated at 37 °C in a humidified atmosphere of 5% CO_2_. When the cells reached 80% confluence in culture flasks, trypsin-EDTA was used to remove the cells and the cells were used in experiments or reseeded in flask.

HMEC-1 cells (3 × 10^4^/mL) or 4T1 cells (3 × 10^4^/mL) were seeded in 96-well flat-bottom culture plates with 100 μL culture medium and incubated overnight. Subsequently, 100 μL culture media containing various concentrations (0.0625–1 μM) of actein were added into the wells. Then the plates were incubated at 37 °C for 48 hours. Plain medium containing vehicle solvent was added to the control wells. The effects of actein on the cell viabilities and proliferation of HMEC-1 or 4T1 cells were assessed by MTT and [^3^H]-thymidine incorporation assays, respectively, as described in our previous study^40^. The effects of actein on the viable cell number of HMEC-1 cells were also determined by trypan blue assay.

### Cell migration assay

A modified Boyden chamber assay was used to assess the cell migration ability of HMEC-1 or 4T1 cells^40^. Briefly, cells (3 × 10^4^ in 100 μL medium) were added into each Transwell inserts. At the same time, 100 μL of medium containing various concentrations of actein (with 1% v/v FBS) was added to the upper chambers. Five hundred microliters of medium (with 10% v/v FBS) served as chemoattractant media was added to the lower chambers. The migrated cells were quantified by manual counting in blinded fashion. The change in cell number is represented as a percentage of control.

The cell motility of HMEC-1 and 4T1 cells was also evaluated using scratch wound assay as described previously^39^,^40^. In brief, cells (1 × 10^5^ in 1 mL medium) seeded in the wells of 24-well plate were scraped with a cross. Then the medium was changed with fresh medium with 5–20 μM of actein and cells were incubated for 16 hours. Each well was photographed before and after incubation. The percentages of open wound area were measured and calculated. The changes of open wound area represent the motility of cells across the scratch wound. The lower the motility of cells resulted in greater open wound area.

### Western blot analysis

Human endothelial cells HMEC-1 (1 × 10^6^/mL) were seeded and incubated for 24 or 48 hours to allow attachment. Different concentrations (1.25, 5, or 20 μM) of actein were added to the dishes and incubated for 24 or 48 hours. After treatments, cells were collected, washed and lysed as described previously^40^.

### Mouse matrigel plug model

Male C57BL/6 mice (6 weeks old) were supplied and maintained by Laboratory Animal Service Center, the Chinese University of Hong Kong. Matrigel (500 μL) was mixed with heparin (10 U/mL), VEGF 100 ng/mL with or without actein (30 μg/mL gel) prior to subcutaneous injections into the flanks of mice. After 7 days, the matrigel plugs were removed and photographed. Another group of mice were inoculated with Matrigel (500 μL) mixed with heparin (10 U/mL) and VEGF 100 ng/mL and they were orally administered with actein (5 or 10 mg/kg) for 7 days. The matrigel plugs were also collected and photographed. The hemoglobin content of the matrigel plugs was quantified using Drabkin’s reagent kit. Hemoglobin content was expressed as mg/mg of wet Matrigel plug^53^.

### Mouse mammary tumor-bearing model

Female BALB/c mice (6–8 weeks old) were obtained from and maintained in Laboratory Animal Services Center, the Chinese University of Hong Kong. Mouse syngeneic mammary tumor 4T1 cells (5 × 10^5^ in 100 μL PBS) were subcutaneously inoculated at the mammary fat pad of each mouse. Mice were treated with actein 8 days after tumor cell inoculation and the treatment lasted for 4 weeks. The tumor-bearing mice were randomly assigned into 3 groups (n = 12): vehicle control group, actein 10 mg/kg and actein 15 mg/kg groups. The doses of actein were chosen after the dose-finding pilot study. Actein was dissolved in DMSO and diluted in distilled water and orally administered to mice daily. The vehicle control group received the vehicle solvent (0.5% v/v DMSO) in distilled water. The positive control group was treated with doxorubicin (5 mg/kg) once a week for 4 weeks. During treatments, the body weight and tumor size of each mouse were measured twice a week. At the end of experiment, mice were sacrificed under anesthesia. The lungs and livers were removed for quantification of tumor burden and spleens were excised for lymphocyte isolation^54^,^55^. Tumors of mice from different groups were also excised for immunohistochemical and molecular analysis.

All experimental methods in mice were carried out in accordance with the approved guidelines specified by the Animal Experimentation Ethics Committee of the Chinese University of Hong Kong. All experimental protocols were approved by the Animal Experimentation Ethics Committee of CUHK with reference numbers Ref No. 10/013/MIS and 10/051/MIS.

### Histological and immunohistochemical analysis

Tumors, lungs and livers were fixed in 10% buffered formalin and then paraffin embedded, sectioned at 5 μm. The sections of lung and liver were stained with hematoxylin & eosin and examined as described previously^55^. Tumor burden, defined as the tumor area, was calculated from the section of the lung or liver and expressed as an average percentage of tumor area to lung or liver area in each treatment group.

The level of cell apoptosis in tumor sections was determined with TUNEL assay using *in situ* cell death POD kit. The assay was carried out according to the procedures recommended in the assay kit manual. The tumor sections were also stained with anti-mouse CD34 and Factor VIII antibodies using an immunohistochemical method as described in previous studies^41^,^53^,^56^,^57^. Four fields of tumor sections were randomly selected, the CD34 and Factor VIII stained endothelial cells or capillaries in brown were considered as positive and the brown area was quantified in the photos using Image J software (NIH, USA).

### Real time-PCR analysis

The tumors excised from actein-treated or untreated tumor-bearing mice were snap frozen in liquid nitrogen. The total RNA of each tumor was extracted, quantified and subjected to reverse transcription as described previously^53^. To quantify the amount of mRNA of *VEGFR1, VEGFR2, Ang1, Ang2, Tie1, CXCR4, AKT,* RT-PCR were performed in Bio-Rad CFX96™ Real-time system C1000 Thermal cycler using the QuantiFast SYBR Green RT-PCR kit from Qiagen^40^,^53^. The sequences of primers are listed in Table 1. Each 10 μL PCR reaction mix contained 100 ng RNA, 5 μL 2x QuantiFast SYBR Green RT-PCR Master Mix, 0.08 μL QuantiFast RT Mix, RNase-free water and 1 μL of both the specific primers (20 μM, Life Technologies, NY, USA). Reactions were performed using the following protocol: pre-incubation at 50 °C for 10 min, then 95 °C for 5 min followed by 40 PCR cycles at 95 °C for 10 s and 60 °C for 30 s (95 °C for 3s, 60 °C for 30 s, and 72 °C for 5 s). A melt curve analysis was performed at the end of the reaction to assess the specificity of the amplification. Relative quantification was obtained by the comparative threshold cycle (*ΔΔ*Ct) method (CFX Manager Software, version 1.6, Bio-Rad, Hong Kong). The specific gene mRNA levels were normalized to that of the internal control *beta-2 microglobulin (B2M*) and then expressed as the fold change compared to the control group.

### Statistical analysis

Data were expressed as mean + SD (*in vitro*) or mean + SEM (*in vivo*). Statistical analyses and significance were analyzed by one-way ANOVA with Tukey’s *post-hoc* test using GraphPad PRISM software version 6.0 (GraphPad Software, USA). In all comparisons, *p* < 0.05 was considered as statistically significant.

## Additional Information

**How to cite this article**: Yue, G. G.-L. *et al*. New potential beneficial effects of actein, a triterpene glycoside isolated from *Cimicifuga* species, in breast cancer treatment. *Sci. Rep.* **6**, 35263; doi: 10.1038/srep35263 (2016).

## Figures and Tables

**Table 1:** Gene specific PCR primers.

	**Forward primer**	**Reverse primer**
*Ang-1*	5′-ACAGCAGTGGAACCGAACAG-3′	5′-AGCCTCCGCCAGCAGAC-3′
*Ang-2*	5′-AGGTGGAGGCTGGACTGTC-3′	5′-GTGGTGAGCAGGTGGATGAC-3′
*Tie-1*	5′-GCGATGGATGGCTATTGAGTCTCTA-3′	5′-GCATCTATTCCAGCATA-3′
*VEGFR1*	5′-CCAGCAGCGAAAGCTTTGCG-3′	5′-CTCCTTGTAGAAACCGTCAG-3′
*VEGFR2*	5′-GCAGGGGACAGAGGGACTTG-3′	5′-GAGGCCATCGCTGCA CTCA-3′
*m-CXCR4*	5′-TGGAACCGATCAGTGTGAGTA-3′	5′-TGGTGGGCAGGAAGATCCTA-3′
*AKT*	5′-GACAGCATGGAGTGTGTGGA-3′	5′-CTGTGCCACTGGCTGAGTAG-3′
*Beta-2 microglobulin*	5′-TGGTGCTTGTCTCACTGACC-3′	5′-GGATTTCAATGTGAGGCGGG-3′
